# Qualité du système d’information et de suivi des interventions en santé dans les zones exposées au financement basé sur les résultats en 2014 au Bénin

**DOI:** 10.11604/pamj.2017.28.257.10967

**Published:** 2017-11-22

**Authors:** Lamidhi Salami, Edgard-Marius Ouendo, Benjamin Fayomi

**Affiliations:** 1 Département de Politique et Système de Santé de l’Institut Régional de Santé Publique d’Ouidah, Université d’Abomey Calavi, Bénin; 2 Département de Santé Publique de la Faculté des Sciences de la Santé de Cotonou, Université d’Abomey Calavi, Bénin

**Keywords:** Financement basé sur les résultats, FBR, indice de la qualité, système d´information, suivi, Bénin, performance, Performance-based financing, PBF, quality index, information system, effectiveness, Benin, performance

## Abstract

**Introduction:**

Le financement basé sur les résultats (FBR) est une intervention à l’échelle du système de santé dont les effets sur les piliers de ce système ne sont pas souvent mesurés, surtout au niveau du système d’information et de suivi des interventions de santé.

**Méthodes:**

L’étude transversale faite au Bénin dans 67 formations sanitaires tirées au hasard dans deux zones sanitaires FBR_PRPSS, deux zones FBR_PASS, toutes exposées au FBR et deux zones non exposées au FBR, a permis d’évaluer la qualité du système d’information et de suivi. L’indice de qualité et les scores de performance des composantes du système ont été utilisés pour comparer les strates exposées au FBR à la strate non exposée.

**Résultats:**

La qualité du système d’information et de suivi est moyenne dans les trois strates, avec des indices de qualité plus élevés dans les strates FBR_PRPSS (77%) et FBR_PASS (74%) que dans la strate Non_FBR (67%). La distribution de la qualité du système au sein des strates est plus favorable aux strates exposées aux FBR. Les composantes ayant une bonne performance sont “Information démographique”, “Résultats et analyses essentiels” et “Archivage des supports statistiques”. Toutefois, des composantes importantes pour le FBR et le système d’information telles “Supervision” et “Rapportage” continuaient d’avoir un IQ moyen, deux ans après le début de l’intervention.

**Conclusion:**

La performance moyenne du système d’information et de suivi pourrait davantage s’améliorer avec le respect des directives du FBR, surtout si la qualité de ce système devient une priorité du FBR.

## Abstract (en)

**Introduction:**

Performance-based financing (PBF) approach is a public health intervention, whose effects on the pillars of this system are often not measured, especially with regard to the information system and to the effectiveness of public health interventions.

**Methods:**

Our cross-sectional study was conducted in Benin in 67 health units randomly drawn from two PBF_HSS (Health Systems Strengthening) health zones and two PBF_NHSSP (Health Sector Support Program) areas, all experiencing PBF, and from two areas where the PBF had not been implemented. It allowed to evaluate the quality of the information and the effectiveness of public health interventions. The quality index and the performance scores of the system components were used to compare the strata covered by the PBF and the noncovered strata.

**Results:**

The quality of the information system and of the effectiveness of public health interventions was average in the three strata, with a higher quality index in the PBF_HSS (77%) and PBF_NHSSP (74%) strata than in the Non_PBF (67%) strata. Health system quality distribution was more favorable in the strata covered by PBF. The components achieving a good performance were “demographic information”, “results and essential analyzes” and “statistic support archiving”. However, the essential components of the PBF and of the information system were “supervision” and “reporting” that continued to have an average QI two years after the beginning of the intervention.

**Conclusion:**

The average quality of the information system and of the effectiveness of public health interventions could be improved by respecting the instructions of the PBF, especially when the quality of this system becomes a priority for the PBF.

## Introduction

La gestion de l’information sanitaire, l’un des six piliers du système de santé selon l’Organisation Mondiale de la Santé, constitue une préoccupation importante pour les responsables qui lui assignent un rôle important dans le choix et la planification des interventions, le monitorage, le suivi et l’évaluation de santé de celles-ci [1]. Cependant, des défaillances importantes sont notées dans le fonctionnement du système d’information sanitaire de la plupart des pays subsahariens, mettant à mal la qualité des choix d’interventions et l’évaluation de l’impact de celles-ci [2]. Pour la vaccination par exemple, l’appui au renforcement du système de santé de GAVI, tout en produisant une amélioration réelle des performances, a provoqué, selon Lim et al. une augmentation considérable de la différence entre la couverture vaccinale officiellement déclarée et la couverture d'enquête [3].

Au Mozambique, Mavimbe et al ont mis en exergue l'ampleur de la mauvaise qualité des données qui touchait chaque type de vaccin avec une différence entre les feuilles de pointage, les registres du service et les rapports de district [4]. Cette mauvaise qualité des données proviendrait de la faible qualité du système de suivi dont plusieurs composantes seraient défaillantes [4,5]. Ces défaillances sont renforcées par le manque d'intérêt des acteurs pour la qualité des informations et l'absence de mécanisme de recoupement, le tout découlant d'un manque de motivation du personnel de santé [4]. Au Bénin, ces mêmes insuffisances du système d’information ont été également relevées aussi bien dans le domaine de la vaccination [6] que dans tout le système d’information [7]. Selon Glèlè et Nzomukunda, elles s’y traduisent par une mauvaise qualité des données produites [6,7]. Cette situation a renforcé chez les décideurs l’attente d’une amélioration du système par le financement basé sur les résultats (FBR) lancé dans le pays depuis 2012.

En effet, le FBR est un transfert d’argent ou de biens matériels d’un organisme de financement ou autre soutien à un bénéficiaire, basé sur la condition que celui-ci entreprenne une action mesurable ou atteigne une cible de performance prédéterminée. D'importants acquis en matière d'utilisation des services et de la qualité des soins sont attribués au FBR dans les pays où des programmes y afférents ont été mis en œuvre [8-10]. Mais, en dehors des résultats de Fairbrother et al. qui ont rapporté un effet positif de l’incitation financière sur la documentation des données de vaccination [11], les preuves d’effets du FBR sur le système d’information sanitaire sont rares. Au regard des orientations déclinées dans le document de cadrage du FBR au Bénin, cette intervention comporte des mécanismes d’appoint devant induire une transmission à temps des rapports, la responsabilisation des acteurs pour la fiabilité des données transmises, la vérification des données à utiliser pour le calcul des subsides à payer aux formations sanitaires [12]. Il s’agit de dispositions dont le niveau d’application et les effets sur le système de santé sont rarement documentés. Au regard des avantages attribués au FBR dans d’autres domaines, des questionnements subsistent quant à sa contribution à la qualité du système d’information dans les pays où il est mis en œuvre. Autrement dit, il est question de savoir si après deux ans de mise en œuvre du FBR au Bénin, le système d’information et de suivi des interventions en santé est meilleur dans les zones exposées au FBR que dans les zones non exposées. Ces questionnements ont suscité la présente étude dont l’objectif est d’évaluer la qualité du système d'information et de suivi dans les zones exposées aux différents modèles de FBR en 2014. Et de façon spécifique, il s’agissait d’apprécier le niveau de mise en œuvre des différentes composantes du système de suivi des services de santé maternelle dans les zones exposées au FBR par rapport aux zones non exposées.

## Méthodes

### Cadre et méthodes d’étude

### Cadre de l'étude

L’étude s’est déroulée dans les zones sanitaires du Bénin, pays de 114.763 Km^2^ de superficie avec une population estimée à 10.361.057 habitants en 2014. Situé en Afrique de l’Ouest, le Bénin est subdivisé en 12 Départements, 77 Communes, 546 Arrondissements. Au plan sanitaire, le système de santé du Bénin est de type pyramidal avec trois niveaux: le niveau central constitué par le ministère de la santé, ses programmes et les hôpitaux nationaux, le niveau intermédiaire constitué par les DDS, leurs services et les hôpitaux départementaux et le niveau opérationnel représenté par les zones sanitaires. Chaque zone regroupe une à quatre communes. La zone sanitaire est subdivisée en des aires sanitaires qui regroupent des villages ou quartiers. En 2014, le pays comptait six départements sanitaires, 34 zones sanitaires et 577 formations sanitaires publiques complètes [13]. Jusqu’en 2014, seules 13 zones étaient couvertes par le FBR: 5 par le Projet d’Appui au Système de Santé (PASS Sourou) financé par la Coopération Technique Belge et 5 par le Projet de Renforcement de la Performance du Système de Santé (PRPSS) financé par la Banque Mondiale.

Le système d’information sanitaire du Bénin est organisé par échelon autour du Système National d’Information et de Gestion Sanitaire (SNIGS). Autour de ce noyau central gravitent les systèmes spécifiques mis en place par des programmes comme celui du VIH/Sida, du paludisme, de la tuberculose [13]. Les projets FBR qui ciblent les activités de santé maternelle et infantile (SMI) ont également développé leur circuit d’information, moins spécifique, destiné à les alimenter directement en données provenant des structures de soins. Dans tous ces systèmes, la collecte des données est faite à priori au niveau des formations sanitaires dans des registres, cahiers ou fiches de pointage. Elles sont ensuite utilisées pour produire les rapports SNIGS et les rapports FBR. Les rapports SNIGS et les rapports FBR doivent être transmis au bureau de zone avant le 5 du mois suivant. Les rapports saisis sont ensuite transmis en version électronique aux niveaux hiérarchiques supérieurs (niveau intermédiaire et central). Dans les zones FBR_PRPSS, ces données sont directement transférées sur la plateforme du programme FBR [12].

### Méthode de l'étude

#### Design, population d’étude et échantillonnage

L’étude a consisté en une comparaison ici-ailleurs d’observations du système d’information et de suivi des zones exposées au FBR et celles des zones non exposées. Elle s'est déroulée entre Juin et Septembre 2015. L’étude a eu lieu dans les zones sanitaires et a ciblé les formations sanitaires ayant régulièrement offert les services de santé maternelle et infantile (SMI) entre 2011 et 2014. Les zones sanitaires couvertes par l’étude ont été retenues au sein des strates ci-après constituées selon le modèle de FBR: 1) la strate du modèle FBR_PRPSS regroupant les zones où le FBR est développé par le Projet Renforcement de la Performance du Système de Santé (PRPSS) sur financement de la Banque mondiale; 2) la strate du modèle FBR_PASS regroupant les zones sanitaires où le FBR est développé par le Projet d’Appui au Système de Santé (PASS Sourou), sur financement de la Coopération Technique Belge (CTB); 3) la strate Non_FBR regroupant les zones sanitaires où le FBR n'est pas encore développé. Les zones sanitaires ne disposant pas d’hôpital de référence fonctionnel depuis 2011, ont été exclues de l’étude, car l’absence de cette infrastructure réduit leur fonctionnalité et compromet l’atteinte des objectifs de santé maternelle et infantile visés par le FBR.

Les critères tels que le nombre de communes, la situation géographique (nord, sud), la similarité de l’accessibilité (zone déshéritée; zone peu ou non déshéritée), la similarité des antécédents récents d'appui de partenaires, ont été utilisés pour apparier les zones sanitaires des trois strates et ainsi former des trios de zones. Un tirage aléatoire simple a été fait pour retenir les deux trios de zone. Les formations sanitaires ont été retenues par choix aléatoire simple dans chaque zone sanitaire. Les données ont été recueillies auprès des prestataires en charge des activités de SMI, du responsable de la formation sanitaire ou du gestionnaire des données. L’outil utilisé pour cette étude provient d’une adaptation de l’outil d'auto-évaluation de la qualité des données de vaccination (DQS) conçu par l'OMS [14]. Les techniques de collecte utilisées sont l’entretien individuel avec les prestataires, accompagné de l’observation et du dépouillement des supports de gestion des formations sanitaires. Les aspects du système d’information et de suivi des activités SMI abordés par cette étude ont été regroupés en sept composantes. Chaque aspect des composantes a été coté entre 1 et 3 points selon son poids. La cote est attribuée dans une formation sanitaire lorsque la variable est présente.

La synthèse des cotes obtenues a permis d’établir le score de performance (SP) de chaque composante et l’indice de qualité (IQ), principale mesure de la qualité du système d’information et de suivi des activités de santé maternelle et infantile (SMI). L’IQ est calculé en faisant le ratio entre la somme des scores obtenus par composante du système et la somme des scores possibles. Les mêmes seuils d’appréciation ont été utilisés pour le système et ses composantes. Ils sont dits: 1) bons ou performants si IQ ou le SP ≤ 80%; 2) moyens si 50% ≥ IQ ou le SP < 80%; 3) mauvais ou médiocres si IQ ou le SP < 50%.

#### Analyse et interprétation des données

Les données collectées ont été saisies dans un masque conçu en Excel. L’indice de qualité du système a été calculé globalement par strate, puis par formation sanitaire pour en saisir la distribution. La comparaison des strates exposées au FBR à la strate non exposée, prise pour référence, a été faite en calculant les différences de proportion, avec leurs intervalles de confiance à 95%, pour les IQ et les scores de performance des composantes. Le test de khi2 réalisé a été apprécié au seuil p-valeur < 5%.

#### Considérations éthiques

Le protocole a été validé par le comité d’éthique. Les autorisations administratives et techniques ont été obtenues auprès des autorités du ministère de la santé et des projets de mise en œuvre du FBR.

## Résultats

### Caractéristiques des formations sanitaires couvertes par l'étude

Les 67 formations sanitaires ayant participé à l’étude, sur l’effectif de 107 que comptaient les zones sanitaires sélectionnées, avaient régulièrement conduit les activités de SMI. Elles ont enregistré 124.199 actes dans leur base logiSNIGS pour le compte des premiers semestres 2011 et 2014.

### Distribution des formations sanitaires selon leur indice de qualité

**Figure 1:**
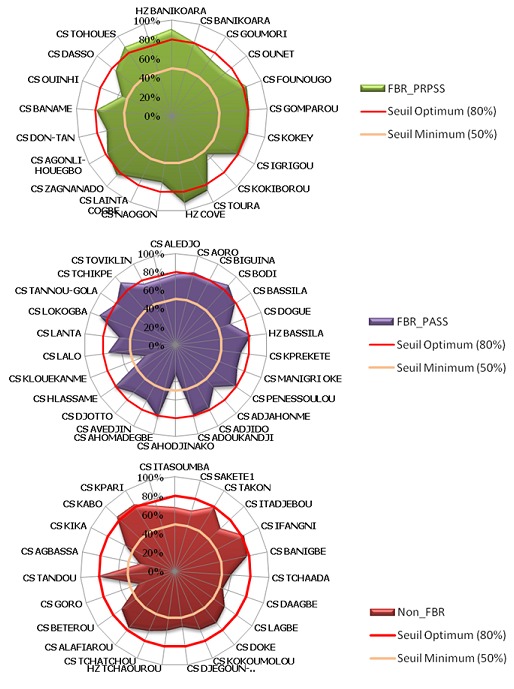
Indice de qualité du système de suivi des interventions en santé maternelle et infantile dans les formations sanitaires des trois strates Non_FBR, FBR_PASS et FBR_PRPSS au Bénin en 2014

La Figure 1 présente la distribution de l’IQ des formations sanitaires des trois strates en 2014. Dans la strate FBR_PRPSS, l’indice de qualité dans les formations sanitaires enquêtées variait entre 55% et 92%. Le mode était à 84% et la médiane de 79%. Dans la strate FBR_PASS, l’intervalle de variation est plus grand, soit 33% à 91%, avec une médiane située à 78% et le mode à 80%. Dans la strate Non_FBR, la plus faible performance était de 38% et la plus élevée 84%. La performance médiane se situait à 68% pour un mode à 82%. Au total, selon le Tableau 1, la moitié des formations sanitaires de la strate FBR_PRPSS avait un bon système de suivi (indice de qualité IQ ≥ 80%). La strate FBR_PASS comptait 28% de formations sanitaires à bon système de suivi contre 18% pour la strate Non_FBR. Les formations sanitaires ayant de mauvais système étaient au nombre de deux dans la strate FBR_PASS et deux dans la strate Non_FBR.

**Tableau 1:** Répartition des formations sanitaires enquêtées dans les trois strates selon la qualité de leur système d’information et de suivi en 2014, Bénin

		Effectif	Proportion
Strate non FBR	(n=22)		
	Bon système	4	18%
	Système moyen	16	73%
	Système mauvais	2	9%
Strate FBR_PASS	(n=25)		
	Bon système	7	28%
	Système moyen	16	64%
	Système mauvais	2	8%
Strate FBR_PRPSS	(n=20)		
	Bon système	10	50%
	Système moyen	10	50%
	Système mauvais	0	0%

NB: IQ ≥ 80%: Bon système; 50% ≤ IQ <80%: système moyen; IQ < 50%: système mauvais

### Qualité globale du système de suivi et niveau de performance des composantes du système

En 2014, le système d’information et de suivi des services de santé maternelle et infantile dans la zone Non_FBR présentait un niveau d’exécution des composantes “Informations démographiques” et “rapportage” à plus de 80%. Dans le même temps, “archivage des supports statistiques” était faible (38%). Les autres composantes étant moyennement exécutées, cette strate affichait globalement une performance moyenne avec un IQ de 67%. Dans la strate FBR_PASS, en dehors de la composante “Informations démographiques” qui était bien exécutée (99%), toutes les autres composantes du suivi ont présenté des scores de performance moyens, allant de 56% pour la “Retro-information” à 79% pour “Résultats et analyses essentielles”. La performance globale du système dans cette strate était de 74%.

Deux composantes du suivi étaient correctement exécutées dans la strate FBR_PRPSS en 2014. Il s’agit de “Informations démographiques” et des “Résultats et analyses essentiels”. Les cinq autres composantes restantes étaient moyennement mises en œuvre, avec des scores variant entre 65% (“rapportage” et “supervision formative”) et 74% (“enregistrement”). L’indice de qualité (IQ) était de 77% dans cette strate.

### Comparaison de la qualité du système de suivi des services des trois strates

En comparant l’indice de qualité des différentes strates en 2014, il ressort que les strates exposées au FBR avaient un système d’information et de suivi de meilleure qualité. Par rapport à la strate Non_FBR, elles présentaient un indice de qualité significativement plus élevé d’environ 10% et 6% respectivement pour la strate FBR_PRPSS et la strate FBR_PASS (Tableau 2). Ces deux strates avaient également des scores de performance (SP) plus élevés que ceux de la strate Non_FBR pour les composantes “Informations démographiques” (plus de 15%), “Résultats et analyses essentiels” (plus de 25%) et “Archivage des supports statistiques” (plus de 23%) (p < 0,001). Ces différences du score de performance observées pour les trois composantes n’étaient pas statistiquement différents d’une strate FBR à l’autre. En ce qui concerne les composantes “Enregistrement” et “Supervision formative”, les différences notées n’étaient pas significatives (p > 0,05).

**Tableau 2:** Comparaison de l’indice de qualité et des scores de performance des composantes du système d’information et de suivi des strates exposées au FBR à ceux de la strate Non_FBR en 2014

Composantes / IQ	Strates	Effectif N	Score n (%)	Différence de proportion (%	IC 95% (%)
Informations Démographiques	FBR_PRPSS	200	197 (99%)	14,86^+^	[9,69; 20,03]
	FBR_PASS	250	248 (99%)	15,56^+^	[10,55; 20,58]
	Non_FBR	220	184 (84%)		
Enregistrement	FBR_PRPSS	260	193 (74%)	3,25^+++^	[-4,23; 10,73]
	FBR_PASS	325	234 (72%)	1,02^+++^	[-6,16; 8,20]
	Non_FBR	286	203 (71%)		
Rapportage	FBR_PRPSS	208	135 (65%)	-15,67^+^	[-23,85; -7,49]
	FBR_PASS	263	201 (76%)	-4,15^+++^	[-11,31; 3,00]
	Non_FBR	242	195 (81%)		
Résultats et analyses essentiels	FBR_PRPSS	337	292 (87%)	32,90^+^	[26,68; 39,13]
	FBR_PASS	425	337 (79%)	25,55^+^	[19,20; 31,91]
	Non_FBR	374	201 (54%)		
Supervision formative	FBR_PRPSS	231	150 (65%)	-0,22^+++^	[-8,64; 8,20]
	FBR_PASS	280	160 (57%)	-8,01^+++^	[-16,17; 0,15]
	Non_FBR	264	172 (65%)		
Retro-information	FBR_PRPSS	118	87 (74%)	-0,51^+++^	[-11,41; 10,38]
	FBR_PASS	150	84 (56%)	-18,24%^++^	[-29,14; -7,34]
	Non_FBR	132	98 (74%)		
Archivage des supports statistiques	FBR_PRPSS	98	65 (66%)	28,14^+^	[15,11; 41,18]
	FBR_PASS	125	76 (61%)	22,62^+^	[10,14; 35,10]
	Non_FBR	110	42 (38%)		
Indice de qualité	FBR_PRPSS	1452	1119 (77%)	9,81^+^	[6,66; 12,95]
	FBR_PASS	1818	1340 (74%)	6,45^+^	[3,40; 9,50]
	Non_FBR	1628	1095 (67%)		

^+^: p-valeur<0,001

^++^: p-valeur<0,05

^+++^: p-valeur>0,05

n = nombre total de points possibles N = nombre de points obtenus dans la strate

## Discussion

Au cours de cette étude, toutes les structures retenues ont été visitées et les données y ont été collectées. La qualité du système d’information et de suivi était moyenne dans toutes les strates, avec des IQ variant entre 67% et 77%. Cette performance comporte des disparités favorables aux strates exposées au FBR, car la moyenne des indices de qualité (IQ) y était plus élevée. Cette performance est pareille à celle constatée en Côte d’Ivoire, où 93% des districts sanitaires et 50% des centres de santé avaient, en 2012, un IQ compris entre 50 et 80% pour le système d’information et de suivi de la vaccination [15]. Les IQ de notre étude étaient plus élevés que la moyenne des IQ du système de suivi de la vaccination de vingt sept pays ciblés par Ronveaux et al en 2003 [5]. Ces derniers, en évaluant cinq composantes du système de vaccination, à savoir l'enregistrement, le stockage et le rapportage, le suivi et l'évaluation, les dénominateurs et la conception du système, avaient retrouvé un IQ de 63% (intervalle 15-97%) au niveau district. Les résultats de Nzomukunda qui a trouvé un IQ moyen de 55% dans les centres de santé de Kandi, ainsi que ceux de Bosch-Capblanch et al. soit un IQ médian de 3,1 sur 5,0 dans 912 services de soins, sont également plus bas que les résultats de notre étude [6,16].

Toutefois, cette relative bonne performance dans les trois strates est empreinte de disparité de l’IQ. En 2014, cette disparité se traduisait par une variation de la distribution de la bonne qualité du système d’une strate à l’autre. Les formations sanitaires ayant un bon système de suivi (indice de qualité IQ ≥ 80%) étaient plus nombreuses dans les strates exposées au FBR, soit 28% pour la strate FBR_PASS et 50% pour la strate FBR_PRPSS, que dans la strate Non_FBR (18%). Le constat était inverse pour les formations sanitaires ayant un système de suivi médiocre (IQ < 50%).

En comparant le niveau du système de suivi des services de santé maternelle et infantile dans les différentes strates en 2014, il est noté que les strates exposées au FBR disposaient d’un système de suivi de meilleure qualité. Leurs indices de qualité étaient significativement plus élevés d’environ 10% et 6% par rapport à celui de la strate Non_FBR. La composante “Informations démographiques” avait le fort score d’exécution dans toutes les strates. Ce constat est pareil à celui fait par Ronveaux et al. qui avaient enregistré, au cours de leur étude, le meilleur score au niveau de la composante “usage des dénominateurs” [5]. Dans le contexte du FBR, le résultat de cette composante dans notre étude confirme les efforts fournis par les équipes de gestion de l’intervention et les responsables des structures de santé pour répondre aux besoins quasi permanents de données de base essentielles pour la planification, le financement et l’évaluation du FBR. Il s’exprime par l’existence dans la plupart des formations sanitaires exposées aux FBR, de carte sanitaire et/ou de données de population régulièrement actualisées. La mise en œuvre du FBR pourrait également justifier la nette amélioration de la composante “Résultats et analyses essentiels” [12]. Les résultats de cette composante dans les trois strates de notre étude sont meilleurs à ceux de certaines structures sanitaires d’Afrique du sud, où “l’analyse et l’interprétation des données”, ainsi que la “retro information” sont rares. Ce qui témoigne de la culture d’utilisation de l’information essentielle [17].

En ce qui concerne la contribution des différentes composantes à la faible performance du système, Ronveaux et al. observent dans leur étude qu’à tous les niveaux, la plus faible composante était le “Suivi et évaluation” [5]. Ce que corroborent les résultats de Nzomukunda à Kandi [6] et de Vroh et al. en Côte d’Ivoire [15]. Ces derniers indexent également la “supervision et le monitorage” [15]. Dans notre étude, les composantes défaillantes variaient selon les strates. Dans les strates Non_FBR, c’est “l’archivage des supports statistiques” qui était sous exécutée, tandis que pour la strate FBR_PASS, c’était la composante “Retroinformation” (56%), et pour la strate FBR_PRPSS, “Rapportage” et “Supervision formative” (65%) étaient concernés.

Ces constats sur la supervision et le rapportage sont meilleurs à ceux de Ronveaux et al. qui justifiaient l’absence d’exactitude des données notée au cours de leur étude par la rareté des visites de contrôle (menées dans moins de 50% des districts et services de santé) [5]. Dans notre étude, il s’agissait plus de la faible exploitation des résultats des visites de supervision et de la faible mise en œuvre des activités de supervision et contrôle internes. Ces activités internes étaient quasi inexistantes et sans documentation dans la plupart des formations sanitaires. Le suivi des recommandations issues des supervisions et leur transformation en des tâches attribuées aux membres de l’équipe de santé ne se faisaient pas dans les formations sanitaires. Ces insuffisances ont été moins notées dans la strate FBR_PASS où la supervision externe était plus régulière et le suivi de ses recommandations plus constant. Les insuffisances relevées au niveau de la composante “Supervision” relèverait plus de déficit en compétence managériale que d’inaptitude technique. Cela pose le problème global du dispositif d’encadrement, d’accompagnement des prestataires, d’organisation des services et surtout de capitalisation des pratiques dans les structures de santé. Selon les constats faits dans les structures de santé, la faible performance de la composante “Rapportage” résulterait de la non disponibilité dans plusieurs structures de documents traçant la transmission des rapports et donc la promptitude de transmission. Les composantes “Rapportage” et “Supervision” jouent un rôle important dans la validation des données et l’assurance de leur cohérence d’un support à un autre [12]. Leur performance moyenne dans la strate FBR_PRPSS, appelle un regard accru sur la qualité de la mise en œuvre du FBR, notamment en ce qui concerne l’application des directives de validation des données et le suivi des activités y afférentes à tous les niveaux. Après deux ans de mise en œuvre du FBR, le niveau de performance de ces composantes dans les strates FBR oriente vers des besoins de compétence des acteurs chargés de la gestion et l’encadrement des structures de santé et la nécessité d’harmonisation accrue des pratiques.

Par ailleurs, jusqu’en 2014 les équipes techniques de zone sanitaire et du niveau intermédiaire n’étaient pas encore enrôlées dans le processus FBR du modèle FBR_PRPSS. De ce fait, elles ne pouvaient disposer de primes ou subsides pour mener les activités de supervision. Cette situation, bien que pouvant contribuer à la lenteur du changement souhaité, ne pourrait, à elle seule, générer les résultats obtenus. L’absence ou le faible écart entre les strates FBR et la strate Non_FBR pour les résultats des composantes “Enregistrement” et “Rapportage” ne semblent pas confirmer l’effet positif de l’incitation financière sur la documentation des services, tel que rapporté par d’autres auteurs [11].

Au Mozambique, en plus des facteurs ci-dessus évoqués, présentés plus sous les thèmes de “manque d'intérêt pour la qualité des informations”, “d'absence de mécanisme de recoupement”, le tout fondé sur un manque de motivation du personnel de santé, il y a aussi l’absence de retro-information [4]. L’absence de motivation des prestataires pour la production de données de meilleure qualité peut également être évoquée dans les zones FBR, tout comme la faible compétence des personnes chargées d’évaluer ou de suivre la qualité des données dans les formations sanitaires des zones FBR. Ces facteurs internes influencent la performance du système, autant que l’existence de mécanismes de décision intégrés et de partage d’expériences, de choix adapté aux connaissances, perceptions et motivations des prestataires [18]. Actuellement, en dehors des trois composantes les plus performantes dans notre étude, qui sont celles les mieux ciblées par certaines directives du FBR, la qualité du système d’information et de suivi ne semble pas être vue comme une priorité de l’approche.

En somme, la meilleure qualité du système d’information et de suivi notée dans les strates exposées au FBR, avec des IQ supérieurs de 6 à 10 points à celui de la strate non exposée, pourrait être associée à la mise en œuvre de l’intervention FBR. Cette qualité pouvait encore être davantage meilleure si les dispositions préconisées par l’intervention étaient entièrement appliquées par un personnel motivé, compétent et bien encadré. Ces conditions suggèrent de prioriser la qualité du système d’information et de suivi dans le FBR et de renforcer le partage d’expériences pour l’harmonisation des pratiques.

## Conclusion

Le système d’information en santé étant un pilier du système de santé, la qualité de ses produits continuera toujours à occuper une place prépondérante dans les préoccupations du système. Les résultats de la présente étude conforte cette assertion en mettant en exergue les difficultés à améliorer la qualité du système de production de l’information en santé. Certes, l’indice de qualité (IQ) est moyen dans les trois strates du pays, mais il est plus élevé dans les strates exposées au FBR. Ce résultat, attribuable à la mise en œuvre du FBR, serait davantage meilleur si la qualité du système d’information était ciblée et achetée directement par le FBR auprès d’un personnel plus qualifié, mieux encadré et dédié à cette activité. Le niveau élevé des scores de performance des trois composantes dont la mise en œuvre est très sollicitée dans le processus FBR confirme qu’une priorisation globale du système de gestion de l’information par le FBR pourrait davantage l’améliorer, en levant tous les goulots en compétence, en organisation et en motivation du personnel. Toutefois, les résultats d’une analyse qualitative du comportement des acteurs devront être pris en compte pour une adéquation des choix.

### Etat des connaissances actuelles sur le sujet

- Les systèmes d’information occupent une place importante dans la performance du système de santé mais leur défaillance est fréquente dans les pays en développement;
- Le financement basé sur les résultats est une intervention majeure qui a amélioré significativement l’utilisation et la qualité des services de santé dans les pays où il est mis en œuvre.

### Contribution de notre étude à la connaissance

- Le financement basé sur les résultats comporte des mécanismes dont la mise en œuvre améliore la qualité du système d’information et de suivi et particulièrement la performance de certaines de ses composantes importantes;
- La qualité du système d’information et de suivi des interventions en santé peut être davantage améliorée si des indicateurs y afférents sont directement achetés par le financement basé sur les résultats.

## Conflits d’intérêts

Les auteurs ne déclarent aucun conflit d'intérêts.

## Remerciements

Nous remercions les autorités du ministère de la santé du Bénin, des projets FBR et de l’Institut Régional de Santé Publique de Ouidah qui ont autorisé la réalisation de cette étude. Cette étude a bénéficié aussi de la subvention African Doctoral Dissertation Research Fellowship (Addrf) accordée par African Population and Health Research Center (APHRC) en partenariat avec International Development Research Center (IDRC).

## References

[1] DAMA International Foundation (2007). Data Management Body of Knowledge. Introduction & Project Status..

[2] Dissongo J Apport de l’information sanitaire au processus de planification des activités dans les structures de santé de la circonscription urbaine de Cotonou 6..

[3] Lim SS, Stein DB, Charrow A, Murray CJ (2008). Tracking progress towards universal childhood immunisation and the impact of global initiatives: a systematic analysis of three-dose diphtheria, tetanus, and pertussis immunisation coverage. Lancet..

[4] Mavimbe JC, Braa J, Bjune G (2005). Assessing immunization data quality from routine reports in Mozambique. BMC Public Health..

[5] Ronveaux O, Rickert D, Hadler S, Groom H, Lloyd J, Bchir A, Birmingham M (2005). The immunization data quality audit: verifying the quality and consistency of immunization monitoring systems. Bull World Health Organ..

[6] Nzomukunda Bété Y (2008). Evaluation de la qualité des données du PEV de routine de l’année 2007 dans la zone sanitaire de Kandi-Gogounou-Sègbana au Bénin..

[7] Glèlè Ahanhanzo Y, Ouendo E-M, Kpozèhouen A, Levêque A, Makoutodé M, Dramaix-Wilmet M (2015). Data quality assessment in the routine health information system: an application of the Lot Quality Assurance Sampling in Benin. Health Policy Plan..

[8] Soeters R, Habineza C, Peerenboom P (2006). Performance-based financing and changing the district health system: experience from Rwanda. Bull World Health Organ..

[9] Basinga P, Gertler P, Binagwaho A, Soucat A, Sturdy J, Vermeersch C Paying primary health centres for performance in Rwanda.

[10] Peabody JW, Florentino J, Shimkhada R, Solon O, Quimbo S (2010). Quality variation and its impact on costs and satisfaction: evidence from the QIDS study. Med Care..

[11] Fairbrother G, Siegel MJ, Friedman S, Kory PD, Butts GC (2001). Impact of financial incentives on documented immunization rates in the inner city: results of a randomized controlled trial. Ambulatory Pediatrics..

[12] Gouvernement du Bénin (2014). Projet de Renforcement de la Performance du Système de Santé. Document de cadrage du financement basé sur les résultats (FBR) au Bénin, version validée..

[13] Ministère de la santé du Bénin (2015). Annuaire des statistiques sanitaires 2014..

[14] World Health Organization (2005). Department of Immunization, Vaccines and Biologicals. The immunization data quality self-assessment (DQS) tool..

[15] Vroh Bénié Bi J, Noufé S, Tiembré I, Bogui YT, Lepri AN, Yohou SK, Walley-Goli C, Dagnan N’Cho S, Saracino TJ (2015). Qualité des données de vaccination chez les enfants de 0 à 11 mois en Côte d’Ivoire. Santé publique..

[16] Bosch-Capblanch X, Ronveaux O, Doyle V, Remedios V, Bchir A (2009). Accuracy and quality of immunization information systems in forty-one low income countries. Tropical Medicine and International Health..

[17] Garrib A, Stoops N, McKenzie A, Dlamini L, Govender T (2008). An evaluation of the District Health Information System in rural South Africa. South African Medical Journal..

[18] Francis Lau, Craig Kuziemsky, Morgan Price, Jesse Gardner (2010). A review on systematic reviews of health information system studies. J Am Med Inform Assoc..

